# PRIMA-1^Met^ suppresses colorectal cancer independent of p53 by targeting MEK

**DOI:** 10.18632/oncotarget.12940

**Published:** 2016-10-27

**Authors:** Tao Lu, Yanmei Zou, Guogang Xu, Jane A. Potter, Garry L. Taylor, Qiuhong Duan, Qin Yang, Huihua Xiong, Hong Qiu, Dawei Ye, Peng Zhang, Shiying Yu, Xianglin Yuan, Feng Zhu, Yihua Wang, Hua Xiong

**Affiliations:** ^1^ Department of Oncology, Tongji Hospital, Huazhong University of Science and Technology, Wuhan, 430030, China; ^2^ Department of Biochemistry and molecular biology, School of Basic Medicine, Huazhong University of Science and Technology, Wuhan, 430030, China; ^3^ Nanlou Respiratory Department, Chinese PLA General Hospital, Beijing, 10083, China; ^4^ BioMedical Research Complex, University of St Andrews, St Andrews, KY16 9ST, UK; ^5^ Department of Pathology, Tongji Hospital, Huazhong University of Science and Technology, Wuhan, 430030, China; ^6^ Biological Sciences, Faculty of Natural and Environmental Sciences, University of Southampton, Southampton SO17 1BJ, UK

**Keywords:** PRIMA-1^Met^, MEK, p53, colorectal cancer, tumorigenesis

## Abstract

PRIMA-1^Met^ is the methylated PRIMA-1 (p53 reactivation and induction of massive apoptosis) and could restore tumor suppressor function of mutant p53 and induce p53 dependent apoptosis in cancer cells harboring mutant p53. However, p53 independent activity of PRIMA-1^Met^ remains elusive. Here we reported that PRIMA-1^Met^ attenuated colorectal cancer cell growth irrespective of p53 status. Kinase profiling revealed that mitogen-activated or extracellular signal-related protein kinase (MEK) might be a potential target of PRIMA-1^Met^. Pull-down binding and ATP competitive assay showed that PRIMA-1^Met^ directly bound MEK *in vitro* and in cells. Furthermore, the direct binding sites of PRIMA-1^Met^ were explored by using a computational docking model. Treatment of colorectal cancer cells with PRIMA-1^Met^ inhibited p53-independent phosphorylation of MEK, which in turn impaired anchorage-independent cell growth in vitro. Moreover, PRIMA-1^Met^ suppressed colorectal cancer growth in xenograft mouse model by inhibiting MEK1 activity.

Taken together, our findings demonstrate a novel p53-independent activity of PRIMA-1^Met^ to inhibit MEK and suppress colorectal cancer growth.

## INTRODUCTION

Colorectal cancer (CRC) is the third most common cancer with nearly 1.4 million new cases in 2012. Based on the lesions within colon or rectum, CRC varies in terms of biological characteristics and carcinogenic mechanisms [1]. Several intracellular signaling pathways such as Ras/Raf/MEK/ERK, PI3k/Akt, Wnt/β-catenin and p53 are frequently dysregulated in CRC [2–4]. Notably, p53 missense or nonsense mutations constantly occur in approximately 40-50% of sporadic CRC, causing therapeutic resistance and poor prognosis [5].

p53 is a key tumor suppressor regarded as the “guardian of the genome”. Once activated in response to a variety of stress, p53 elicits potent inhibition of tumorigenesis, leading to cell cycle arrest, apoptosis, senescence and autophagy [6–8]. However, mutant p53 not only abrogates tumor suppressor functions of wild-type p53 but also acquires an oncogenic “gain-of-function” defined as the ability for malignant proliferation, invasion, metastasis and anti-apoptotic effect [9]. Therefore, a promising strategy to treat cancer with mutant p53 via restoring wild-type p53 function has been pursued. Several small molecules such as PRIMA-1, APR-246 (PRIMA-1^Met^), CP-31398, MIRA have been shown to restore wild-type activity to mutant p53 and induce massive p53-dependent apoptosis [10–13].

PRIMA-1 is one of chemical compounds originally identified by screening a library of low-molecular-weight compounds [10]. PRIMA-1^Met^, as methylated analog of PRIMA-1, could enhance killing efficacy. Biochemical analysis demonstrated that either PRIMA-1 or its methylated version is converted into active product named methylene quinuclidinone (MQ), which in turn covalently binds to several cysteine (Cys) residues in the DNA-binding domains of p53 and restores functional conformation of wild-type p53 [14]. Recent reports revealed other targets of PRIMA-1^Met^ such as oxidosqualene cyclase and selenoprotein thioredoxin reductase 1 (TrxR1) which are sufficient to suppress cancer cell growth irrespective of p53 mutation status [15, 16]. Therefore, identifying potential targets of PRIMA-1^Met^ is important to the development of new cancer therapy approaches.

The mitogen-activated protein kinase (MAPK) pathway Ras/Raf/MEK/ERK is often aberrantly activated in CRC and plays a critical role in multiple cellular processes including proliferation, transformation, apoptosis and senescence [17]. Generally, constitutive activation of MAPK cascades is stimulated by mutant Ras because activating K-Ras mutations occur in approximately 37-45% CRC [18]. So far, no K-Ras mutant inhibitors are available for clinical practice [19, 20]. MEK is a core downstream effector of Ras/Raf kinases and appears as a promising alternative for therapeutic strategy targeting Ras-activated MAPK pathway [21].

In the present study, we reported that PRIMA-1^Met^ attenuated colorectal cancer cell growth irrespective of mutant p53 status. In p53 null or p53 wild-type HCT116 colorectal cancer cells, we demonstrated that PRIMA-1^Met^ effectively inhibited cell proliferation and anchorage independent growth. Furthermore, we showed that PRIMA-1^Met^ directly bound MEK kinase and suppressed its activity both *in vitro* and in cells. Moreover, *in vivo* animal experiments confirmed that PRIMA-1^Met^ inhibited MEK activity to suppress the growth of colorectal cancer xenografts.

## RESULTS

### PRIMA-1^Met^ inhibits the proliferation and growth of CRC cells independent of p53 status

**Figure 1:**
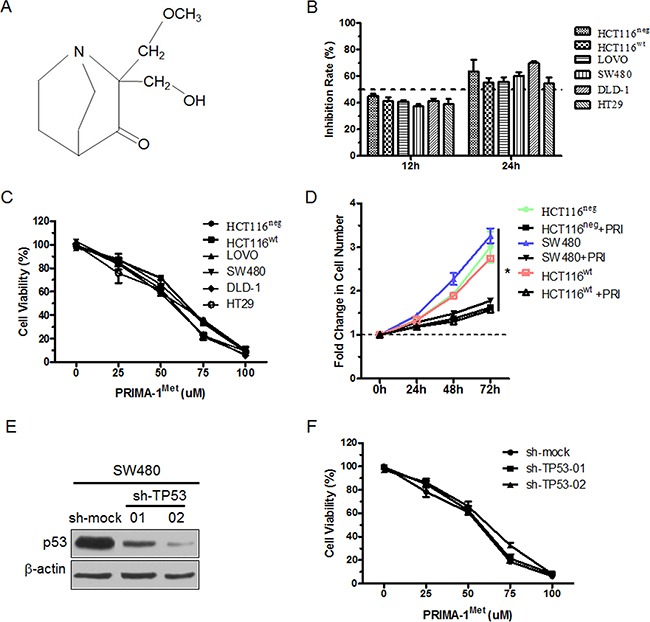
PRIMA-1^Met^ inhibited the proliferation of CRC cells with different p53 status **A.** Chemical structure of PRIMA-1^Met^. **B.** CRC cells with different p53 status were treated with 25 uM PRIMA-1^Met^ for 12 or 24 h. The cell viability was measured by MTS assay. **C.** Six colorectal cancer cells were exposed to PRIMA-1^Met^ with various concentrations for 24 h and cell viability was measured by MTS assay. **D.** 1×10^6^ HCT116^wt^, HCT116^neg^ or SW480 cells were treated with 25 uM PRIMA-1^Met^ or control. Growth curve was measured by counting cell number at indicated time point. *P<0.05. PRI: PRIMA-1^Met^. **E.** SW480 cells were transiently transfected with sh-TP53 or sh-mock and p53 expression was analyzed by Western blot analysis. **F.** SW480 cells transfected with sh-mock or sh-TP53 were treated with various concentrations of PRIMA-1^Met^ for 24 h and cell viability was measured by MTS assay. All data were shown as mean ± SEM from triplicate experiments.

The chemical structure of PRIMA-1^Met^ was shown in Figure 1A. To evaluate p53-independent efficacy of PRIMA-1^Met^, we selected a series of CRC cell lines representative of different TP53 heterogeneity, including TP53^wt^ (HCT116^wt^ and LOVO), TP53^mut^ (SW480, DLD-1 and HT29) and TP53^neg^ (HCT116^neg^). p53 protein expression level and genotypes of these cell lines were presented (Supplementary Figure S1 and Supplementary Table S1). MTS assay showed that the treatment of different cells with 25 uM PRIMA-1^Met^ for 12 h or 24 h led to similar suppression of cell growth, indicating that CRC cells with different TP53 status were generally sensitive to PRIMA-1^Met^ (Figure 1B). Meanwhile, we treated the cells with different concentrations of PRIMA-1^Met^ for 24 h. MTS assay showed that PRIMA-1^Met^ reduced the viability of all six CRC cell lines in a dose-dependent manner (Figure 1C). Notably, the inhibitory effect of PRIMA-1^Met^ at concentrations less than 50 umol/L showed no significant difference among the cells (Figure 1C). However, high dosage of PRIMA-1^Met^ (75 uM) triggered apoptosis, especially in cells harboring mutant TP53 (Supplementary Figure S2A–S2D).

Next we examined the effect of PRIMA-1^Met^ on CRC cell growth, and the results showed that PRIMA-1^Met^ inhibited the growth of three cell lines: TP53^wt^ HCT116^wt^, TP53^mut^ SW480, and TP53^neg^ HCT116^neg^ (Figure 1D), confirming that the inhibitory effect of PRIMA-1^Met^ on CRC cell growth is independent of TP53 status.

To further investigate whether the effect of PRIMA-1^Met^ on cell proliferation is dependent on TP53, we compared the effects of PRIMA-1^Met^ in SW480 or DLD-1 cells transfected with sh-mock or sh-TP53 plasmid (Figure 1E and Supplementary Figure S3A). The viability of sh-mock or sh-TP53 cells exhibited no significant difference (Figure 1F and Supplementary Figure S3B). Taken together, these results suggest that the inhibitory effects of PRIMA-1^Met^ on CRC cell proliferation and growth are independent of p53 status.

### PRIMA-1^Met^ inhibits CRC cell colony formation and EGF-induced cell transformation independent of p53 status

**Figure 2:**
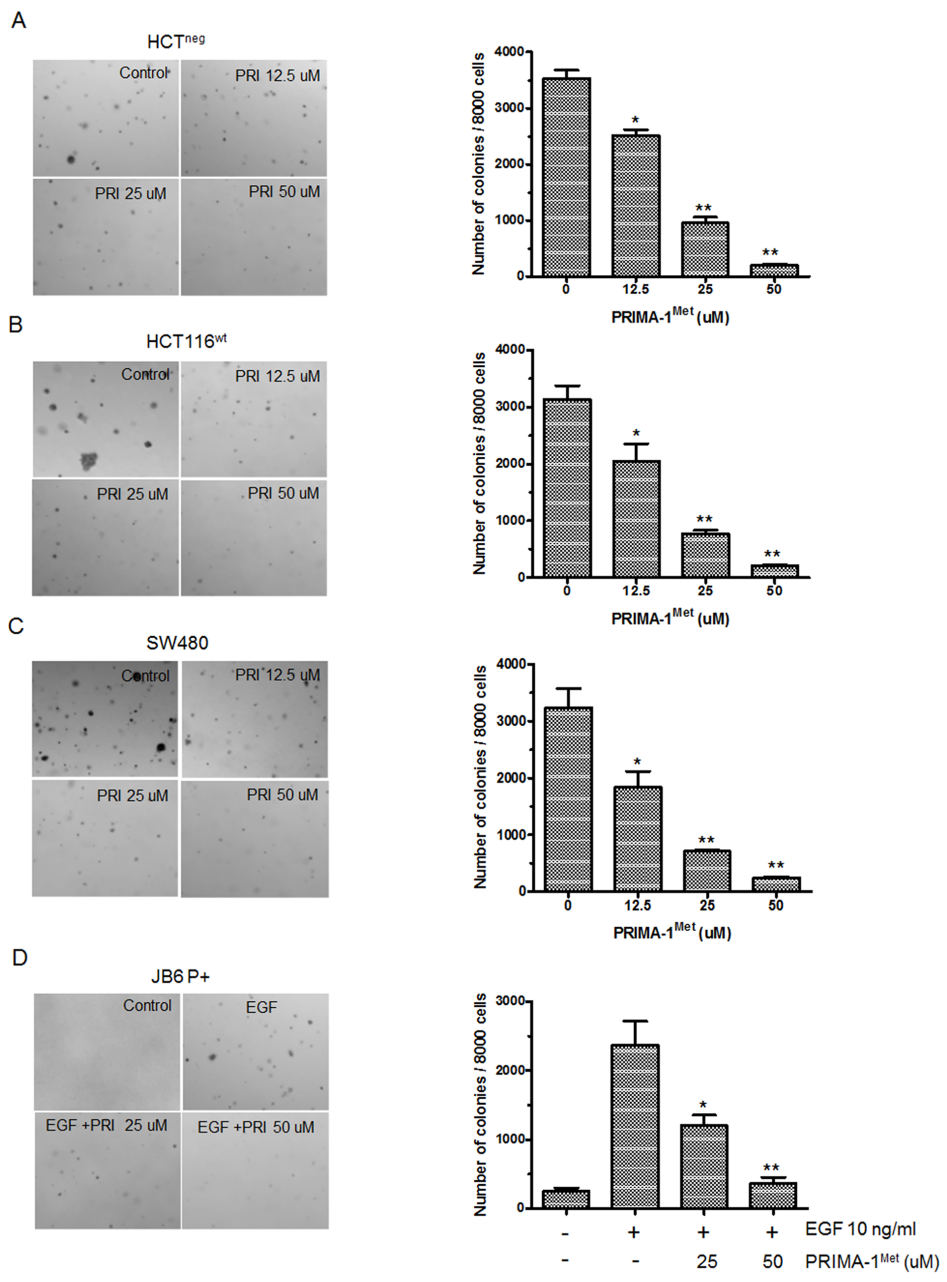
PRIMA-1^Met^ inhibited anchorage-independent growth of colorectal cancer cells and EGF-induced neoplastic transformation of JB6 P+ cells **A-C.**, Representative images of colony formation. Cells were seeded in soft agar with the addition of different concentration of PRIMA-1^Met^ (0, 12.5, 25, 50 uM) and cultured for 7 days (SW480 and HCT116^neg^) or 10 days (HCT116^wt^), then colonies were counted. Data were shown as mean ± SEM from triplicate experiments. ^**^P<0.01 vs. vehicle group. PRI: PRIMA-1^Met^. **D.** JB6 P+ cells were cultured with or without EGF (10 ng/ml) and different concentrations of PRIMA-1^Met^ (0, 25, 50 uM) for 7 days, then colonies were counted. Data were shown as mean ± SEM from triplicate experiments. ^*^ *P*<0.05; ^**^, *P*<0.01 vs. EGF single group. PRI: PRIMA-1^Met^.

Next, we determined the effect of PRIMA-1^Met^ on anchorage-independent growth of cancer cells. Colony formation assay showed that PRIMA-1^Met^ markedly inhibited anchorage-independent growth of HCT116^wt^, SW480 and HCT116^neg^ cells in a concentration dependent manner (Figure 2A–2C). In particular, PRIMA-1^Met^ at 50 uM significantly decreased more than 70% of colony formation in all 3 cell lines compared to control (*P*<0.05). Even at the lower concentration of 25 uM, PRIMA-1^Met^ caused approximate 30% decrease of colony formation in soft agar. Our data showed no significant differences in dose-dependent inhibition by PRIMA-1^Met^ among TP53^wt^, TP53^mut^ and TP53^neg^ cells.

We further accessed whether PRIMA-1^Met^ could inhibit EGF-promoted neoplastic cell transformation in immortalized normal cells. We found that PRIMA-1^Met^ significantly inhibited EGF induced anchorage-independent growth of JB6 P+, an immortalized normal skin cell line with impaired p53 function (Figure 2D). These data strongly suggest that p53 status is not indispensable for PRIMA-1^Met^ mediated inhibition of anchorage-independent growth and EGF-induced cell transformation.

### Kinase profiling identifies PRIMA-1^Met^ as a potential MEK inhibitor

To understand the mode of action of anti-tumor activity of PRIMA-1^Met^, we performed in vitro kinase profiling assay using Millipore's Kinase Profiler (Table 1). The results showed that MEK1 or MEK2 kinase activity was significantly suppressed by PRIMA-1^Met^ at the concentration of 25 uM. At the same concentration, PRIMA-1^Met^ had little effect on EGFR, MAPK1/2 and RSK1/2, which were key kinases in Ras/Raf/MEK/ERK pathway. These data suggest that MEK is a strong candidate target of PRIMA-1^Met^ compared to other screened kinases.

**Table 1:** Kinase profiling results of PRIMA-1^Met^

Kinase	Activity	Kinase	Activity
ALK	90	JNK3(h)	96
AMPKα1(h)	94	KDR(h)	93
Aurora-A(h)	83	MAPK1(h)	75
Aurora-B(h)	91	MAPK2(h)	80
B-Raf (h)	80	MEK1(h)	35
CDK1/cyclinB(h)	94	MEK2(h)	48
CDK2/cyclinA(h)	102	mTOR(h)	86
CDK3/cyclinE(h)	73	p70S6K(h)	90
C-MET(h)	60	PDK1(h)	76
C-RAF(h)	92	PKBα(h)	110
cSRC(h)	67	PKBβ(h)	100
EGFR(h)	95	PKCα(h)	88
FGFR1(h)	83	PKCδ(h)	92
GSK3α(h)	105	Rsk1(h)	88
GSK3β(h)	110	Rsk2(h)	79
IGF-1R(h)	92	Src(T341M)(h)	110
IKKα(h)	94	TGFBR1 (h)	93
IKKβ(h)	90	PI3K (p110α/p85α) (h)	97
JNK1α1(h)	70	PI3K (p110 β/p85α) (h)	90
JNK2α2(h)	84	PI3K (p110δ/p85α) (h)	94

Kinase screening assay was performed according to Millipore's Kinase Profiler protocols. Briefly, the reaction concentration of PRIMA-1^Met^ was set at 100 μM and the concentration of ATP at 10 μM. Scores for each kinase were represented as the percent (%) of kinase activity after PRIMA-1^Met^ treatment.

### PRIMA-1^Met^ inhibits MEK kinase activity *in vitro* and in cells

**Figure 3:**
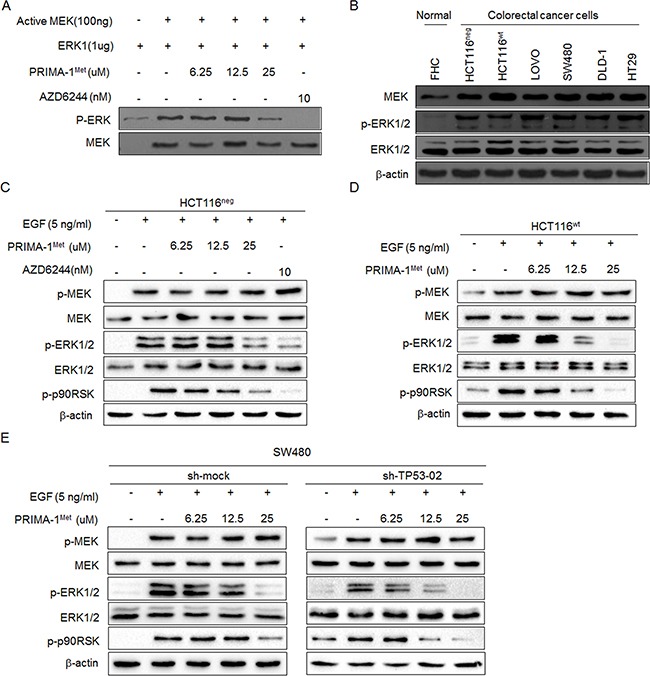
PRIMA-1^Met^ inhibited kinase activity of MEK in vitro and in cells **A.** Representative results of in vitro kinase assay. Inactive ERK1 protein was used as a substrate and mixed with active MEK kinase and different doses of PRIMA-1^Met^. The phosphorylation level of ERK1 (Thr202/Tyr204) was detected by Western blot analysis. Total MEK was used as loading control and MEK inhibitor AZD6244 was used as a positive control. **B.** Western blot analysis of MEK protein expression level in six colorectal cancer cell lines and normal colon epithelial cell line FHC. The activation of MEK signaling was detected in HCT116^neg^ **C.**, HCT116^wt^ **D.** and SW480 **E.** cells. After starvation in serum-free medium for 24 h, cells were treated with different doses of PRIMA-1^Met^ for 12 h and then treated with 5 ng/ml EGF for 30 min. AZD6244 was used as a positive control. Data were representative results from three independent experiments.

To confirm that PRIMA-1^Met^ could inhibit MEK activity, we conducted *in vitro* kinase assay by using inactive ERK1 as a substrate of MEK and MEK inhibitor AZD6244 as a positive control. We found that the phosphorylation of ERK was potently decreased by PRIMA-1^Met^ in a dose dependent manner (Figure 3A).

Next, we detected MEK kinase activity in colorectal cancer cells and found that its activity was significantly elevated in six colorectal cancer cells compared with normal colon cells (Figure 3B). When colon cancer cell lines HCT116^wt^ and HCT116^neg^ were treated with PRIMA-1^Met^, The phosphorylation of downstream molecules of MEK such as ERK1/2 and RSK2 was dramatically attenuated with PRIMA-1^Met^ concentration more than 25 uM (Figure 3C, 3D). However, there were no significant differences in MEK inhibition between HCT116^wt^ and HCT116^neg^ cells, indicating that p53 status does not interfere with PRIMA-1^Met^ induced MEK inactivity.

To further exclude p53-dependent regulatory mechanisms, we depleted the expression of mutant p53 in SW480 cells by shRNA and found that depletion of p53 showed minimal effect on the attenuation of MEK activity after PRIMA-1^Met^ treatment (Figure 3E). Collectively, these data confirm that PRIMA-1^Met^ inhibits MEK activity independent of p53 status.

### PRIMA-1^Met^ binds MEK directly *in vitro* and *ex vivo*

**Figure 4:**
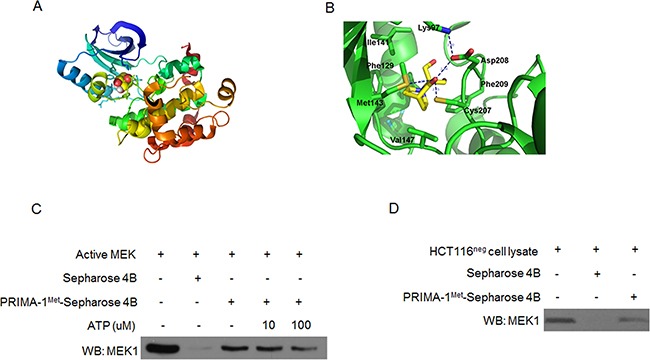
PRIMA-1^Met^ specifically binds MEK *in vitro* and *in vivo* **A.** Computational binding model of PRIMA-1^Met^ in proposed ATP-binding pocket of MEK1 monomer. PRIMA-1^Met^ was shown as colored sphere and MEK1 monomer as cartoon. **B.** The proposed binding-position of hydrogen bonds between PRIMA-1^Met^ and MEK1 was indicated. **C.** Pull-down assay was conducted in vitro by incubating 600 ug HCT116^neg^ cell lysates with PRIMA-1^Met^-Sepharose 4B beads followed by Western blot analysis. **D.** In vivo ATP competitive binding assay was conducted by incubating 300 ng active MEK1 protein with 100 ul PRIMA-1^Met^-Sepharose 4B beads and various concentrations of ATP (0, 10, 100 uM) followed by Western blot analysis. Sepharose 4B only beads were used as negative control. Data shown were representative of three independent experiments.

To better understand PRIMA-1^Met^-induced inhibition of MEK activity, we wondered whether PRIMA-1^Met^ could interact with MEK. We docked PRIMA-1^Met^ to MEK monomer using automated docking tool AutoDock 4 [22]. The results showed that PRIMA-1^Met^ bound to the ATP-binding pocket of MEK1 monomer (Figure 4A). The oxygen atom of PRIMA-1^Met^ could form hydrogen bond with the sulfur group of Cys207 and Met143, and the carboxylate group of Asp208 (Figure 4B).

Next we performed ATP competitive binding assay by using PRIMA-1^Met^-conjugated Sepharose 4B beads *in vitro*. Compared with Sepharose 4B beads alone, PRIMA-1^Met^-conjugated Sepharose 4B beads could pull-down MEK and their affinity was impaired by the presence of ATP (Figure 4C). Furthermore, immunoprecipitation assay in HCT116^neg^ cells confirmed the binding of PRIMA-1^Met^ to endogenous MEK (Figure 4D). Collectively, these data provide strong evidence that PRIMA-1^Met^ could directly bind MEK in ATP-binding pocket *in vitro* and in cells.

### MEK is indispensable for the anti-tumor activity of PRIMA-1^Met^ *in vitro*

**Figure 5:**
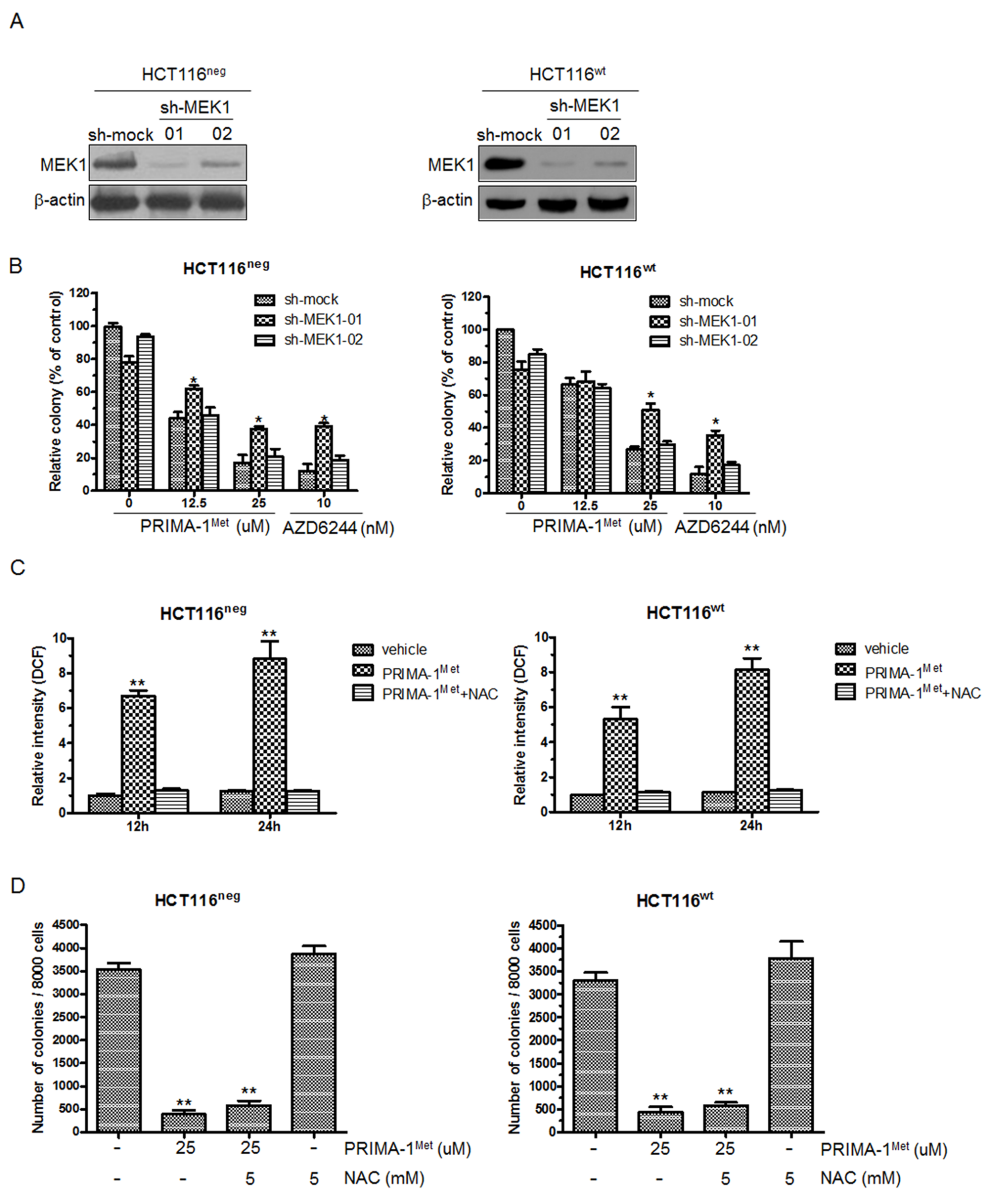
Knockdown of MEK1 decreases the sensitivity of colorectal cancer cells to PRIMA-1^Met^ **A.** HCT116^neg^ or HCT116^wt^ cells were transfected with sh-mock or sh-MEK1. Knockdown efficiency was evaluated by Western blot analysis. **B.** HCT116^neg^ or HCT116^wt^ cells transfected with sh-mock or sh-MEK1 were treated with 12.5 and 25 uM PRIMA-1^Met^ for 7 days and colony formation in soft agar was assessed. Data were shown as mean ± SEM. AZD6244 was used as a positive control. *P<0.05. **C.** HCT116^neg^ cells were treated with 25 uM PRIMA-1^Met^ in the presence or absence of NAC (5 mM) for 12 or 24 h and stained with 20 uM DCFHDA for 30 min. The mean fluorescent intensity was measured by flow cytometer and normalized to vehicle group. Data were shown as mean ± SEM from triplicate experiments. **P<0.01 vs. vehicle group. **D.** Cells were seeded in soft agar with the addition of 25 uM PRIMA-1^Met^ alone or in combination of 5mM NAC and cultured for 7 days. Then colony formation was counted. Data represented mean ± SEM. **P<0.01 vs. control.

To confirm that PRIMA-1^Met^ attenuated cancer cell proliferation and anchorage-independent growth via the inhibition of MEK kinase activity, we used shRNA to knockdown MEK1 expression in HCT116^neg^ or HCT116^wt^ cells (Figure 5A). The depletion of MEK abrogated PRIMA-1^Met^-induced suppression of anchorage-independent growth of HCT116^neg^ and HCT116^wt^ cells, and the magnitude of growth reduction was correlated with the efficiency of MEK1 knockdown (Figure 5B).

PRIMA-1^Met^ could induce reactive oxygen species (ROS) to mediate p53-independent cell apoptosis [16]. Thus it is possible that PRIMA-1^Met^ inhibits CRC cell growth by promoting ROS-induced cell apoptosis. To test this possibility, we measured endogenous ROS in HCT116^neg^ and HCT116^wt^ cells treated with 25 uM PRIMA-1^Met^. PRIMA-1^Met^ induced massive production of ROS and this was significantly inhibited by ROS scavenger NAC (Figure 5C). However, NAC had little effect on PRIMA-1^Met^ induced attenuation of anchorage-independent growth of HCT116^neg^ and HCT116^wt^ cells (Figure 5D). These results indicate that MEK but not ROS predominantly contributes to p53-independent anti-tumor activity of PRIMA-1^Met^.

### PRIMA-1^Met^ suppresses CRC growth in xenograft mouse model by inhibiting MEK activity

**Figure 6:**
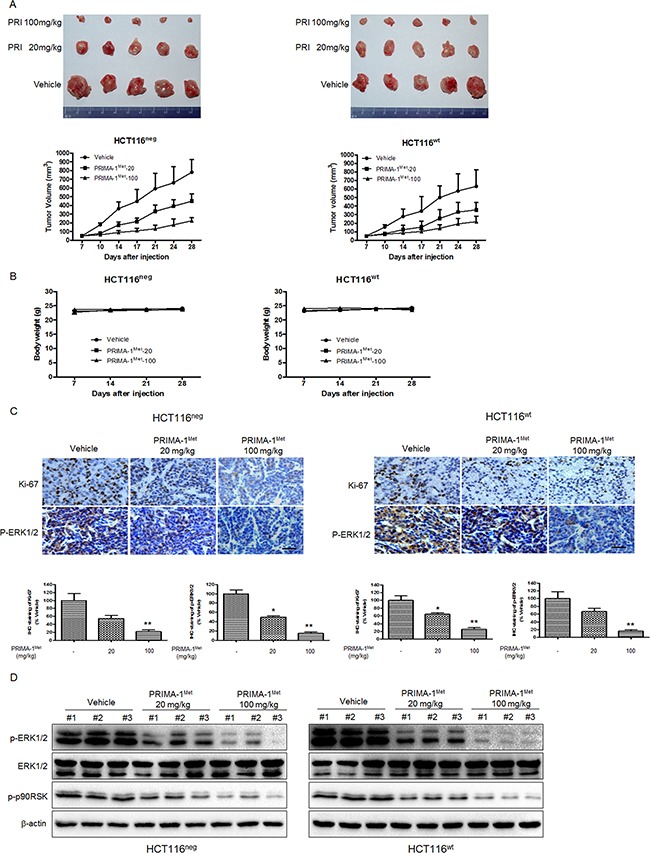
PRIMA-1^Met^ suppresses tumor growth *in vivo* by inhibiting MEK activity **A.** Mice bearing HCT116^neg^ or HCT116^wt^ tumors were treated with PBS, PRIMA-1^Met^ (20 mg/kg or 100 mg/kg). Tumor volume was measured twice a week and data were represented as mean ± SEM. *P<0.05; **P<0.01 vs. vehicle group. **B.** The body weight of mice in each group was measured and recorded once a week. Data were shown as mean ± SEM. **C.** Immunohistochemical staining of Ki-67 and phosphorylated ERK1/2 in paraffin embedded sections of tumor xenografts. Scale bar: 50 um. ^*^P<0.05; ^**^P<0.01. **D.** The levels ofp-ERK1/2, T-ERK1/2 and p-p90RSK in the dissected xenografts were determined by Western blot analysis.

To confirm that the anti-tumor activity of PRIMA-1^Met^ is mediated by MEK *in vivo*, we established subcutaneous xenograft nude mice model implanted with HCT116^neg^ or HCT116^wt^ cells. Once the tumor xenografts grew to approximately 50 mm^3^, the mice were randomly assigned to three groups: vehicle (treated with PBS); lower dose of PRIMA-1^Met^ (20 mg/kg); and higher dose of PRIMA-1^Met^ (100 mg/kg). PRIMA-1^Met^ markedly reduced tumor size in both HCT116^neg^ and HCT116^wt^ derived xenografts in a dose dependent manner (Figure 6A). Compared to vehicle group, PRIMA-1^Met^ (20 mg/kg or 100 mg/kg) significantly inhibited average tumor volume (*P* < 0.05). In addition, body weight was not significantly affected by PRIMA-1^Met^ (Figure 6B), indicating that treatment dose of PRIMA-1^Met^ was well tolerated by the mice.

Immunohistochemical analysis of tumor samples showed that the phosphorylation of ERK1/2 was substantially reduced in treatment groups compared to vehicle group. Additionally, PRIMA-1^Met^ inhibited HCT116^neg^ and HCT116^wt^ cell proliferation because fewer cells were positively stained with proliferation marker Ki-67 compared to vehicle group (Figure 6C). Western blot analysis of tumor samples showed that the phosphorylation of ERK1/2 was substantially suppressed in treatment groups compared to vehicle group in a dose dependent manner (Figure 6D). Taken together, our data indicate that PRIMA-1^Met^ inhibits CRC growth in vivo independent of p53 status by suppressing MEK activity.

## DISCUSSION

Tumor suppressor p53 is encoded by TP53 gene which is frequently mutated in half of all human cancers, including breast cancer, multiple myeloma, lung cancer and colorectal cancer [23]. The most mutations in TP53 such as Y220C or R175H are clustered within the DNA-binding domain and thus impair transcription activity of p53, resulting in the inactivation of p53 targets such as p21, Bax, PUMA and Noxa [24]. As a consequence, loss of p53 tumor suppressor activity prevents cancer cells from p53 mediated cell senescence, cell cycle arrest and apoptosis. Moreover, mutant p53 acquires gain-of-function to promote malignant phenotypes of cancer [25]. Thus it is urgent to develop novel anti-cancer strategies targeting mutant p53.

PRIMA-1 and its methylated form PRIMA-1^Met^ were originally isolated from the low-molecular-weight compounds library for the ability to restore sequence-specific DNA binding and transcription-dependent apoptotic function to mutant p53 *in vitro* and *in vivo* [10]. Although PRIMA-1^Met^ has demonstrated preferential inhibitory effect on cancer cells harboring mutant p53, recent studies suggest p53-independent activity of PRIMA-1^Met^. Saha et al. found that PRIMA-1^Met^ induced apoptosis via p53-independent Noxa induction [26]. Grinter et al. found that PRIMA-1^Met^ exerted anti-cancer effect by the inhibition of oxidosqualene cyclase (OSC), a key enzyme involved in cholesterol synthetic pathway [15]. Because p53 family members p63 and p73 have similar sequence homology to p53 in DNA binding domains, PRIMA-1^Met^ was found to restore mutant p63 and p73 with apoptotic function irrespective of p53 status [27]. Meanwhile, PRIMA-1^Met^ could induce ROS generation and cause p53-independent massive cell death by converting TrxR1 to NADPH oxidase or impairing GSH metabolism [16, 28]. These observations prompted us to evaluate p53 independent ant-tumor activities of PRIMA-1^Met^.

In the present study, we first used PRIMA-1^Met^ to treat six colorectal cancer cell lines with different p53 status. We found that PRIMA-1^Met^ inhibited the proliferation and growthof all cell lines in a dose and time dependent manner, and high dose of PRIMA-1^Met^ triggered cell apoptosis regardless of p53 status. In addition, we found that treatment with 25 or 50 uM PRIMA-1^Met^ caused G2/M arrest in HCT116^neg^ cells (Supplementary Figure S4), consistent with previous study [29]. Moreover, SW480 and DLD-1 cell lines with depleted mutant p53 were as sensitive as their parental cells to PRIMA-1^Met^ treatment, demonstrating that PRIMA-1^Met^ could suppress CRC cell growth in a p53-independent manner.

Kinase signaling pathways, such as Ras/Raf/MEK and PI3-K/Akt/mTOR cascades, are generally hyperactivated and contribute to cancer cell proliferation, migration, invasion and chemoresistance [30]. Therefore, we performed kinase profiling assay by screening total 40 kinases and identified MEK kinase as a potential target that mediates p53-independent anti-tumor activity of PRIMA-1^Met^. MEK kinase and its downstream effectors are consistently activated owing to aberrant mutant activation of Ras and B-Raf in colorectal cancer [31]. Once activated, MEK1 and MEK2 form heterodimer kinase and consecutively mediate downstream ERK1/ERK2 phosphorylation. Several selective and allosteric MEK inhibitors that bind non-ATP competitive site, such as Trametinib, Cobimetinib, MEK162, AZD6244 and PD-0325901, have shown promise in preclinical and clinical studies [32–34]. In contrast, we speculated that PRIMA-1^Met^ directly binds MEK in the ATP-binding pocket based on the results of automated docking analysis, pull-down binding and ATP competitive assay. Thus the mode of action of PRIMA-1^Met^ in inhibiting MEK activity seems to be different from that of MEK inhibitors binding non-ATP competitive site. Further studies are needed to compare the efficacy of PRIMA-1^Met^ and other MEK inhibitors.

By detecting the phosphorylation of MEK substrate ERK, we showed that PRIMA-1^Met^ potently inhibited MEK kinase activity in CRC cells in a dose dependent manner. More importantly, knockdown of mutant p53 in SW480 did not affect the inhibition of MEK activity by PRIMA-1^Met^, confirming that PRIMA-1^Met^ could inhibit MEK kinase activity in cells with different p53 status. Moreover, *in vivo* study showed that PRIMA-1^Met^ inhibited MEK activity, leading to significant tumor regression in HCT116^neg^ and HCT116^wt^ xenograft model compared with vehicle group. These *in vitro* and *in vivo* data indicate that PRIMA-1^Met^ is a novel MEK1 inhibitor.

In this study we depleted endogenous MEK1 in CRC cells to investigate whether MEK1 is indispensable for anti-tumor activity of PRIMA-1^Met^ since many small molecules that targeting the main target have off targets [35, 36]. As expected, the depletion of MEK1 abrogated PRIMA-1^Met^ induced suppression of CRC cell growth. To avoid the confounding potential of ROS, which could be induced by PRIMA-1^Met^ and mediate p53-independent cancer cell apoptosis [16], we further assessed ROS level and found that ROS production was induced after PRIMA-1^Met^ treatment. However, ROS scavenger NAC only slightly compensated for PRIMA-1^Met^ induced CRC cell growth suppression. Collectively, these results demonstrate that direct binding to MEK and inhibiting its activity is the main, if not exclusive, mechanism by which PRIMA-1^Met^ suppresses CRC independent of p53 status.

In conclusion, we provide the first lines of evidence that PRIMA-1^Met^ inhibits colorectal cancer cell proliferation and tumor growth predominately by directly targeting MEK in p53 independent manner. Consequently, targeting both MEK and mutant p53 by PRIMA-1^Met^ may allow us to initiate new strategy for the treatment of malignant colorectal cancer that harbors both hyper-activated Ras/Raf/MEK signaling and p53 mutation.

## MATERIALS AND METHODS

### Cell culture and transfection

HCT116^neg^, HCT116^wt^, LOVO, DLD-1, SW480, HT-29 and JB6 P+ mouse skin epidermal cells were obtained from American Type Culture Collection (ATCC). HCT116^neg^, HCT116^wt^ and HT-29 cells were cultured in McCoy's 5a and other colorectal cancer cells were cultured in RPMI-1640 supplemented with 10% fetal bovine serum (FBS) and 1% antibiotic. JB6+t mouse skin epidermal cells were cultured in Minimum Essential Medium (MEM) supplemented with 5% FBS and 1% antibiotic. Cells were cultured at 37°C in a 5% CO2 incubator. Lentivirus shRNAs pLKO.1-sh-mock, pLKO.1-sh-p53 (#1:TRCN0000003756, #2:TRCN0000003758) and pLKO.1-sh-MEK (#1:TRCN0000002314, #2:TRCN0000002315) were ordered from Thermo Scientific (Huntsville, AL, USA), and co-transfected with packaging vectors (pMD2.0G and psPAX from Addgene) into HEK-293T package cells by using Lipofectamine 2000 (Thermo Scientific, MA, USA). 36 hours after transfection, cell supernatants were harvested, filtered and used to infect target cells. 24 hours after infection, cells were supplemented with 8 ug/ml puromycin to select stable cell lines and RNAi efficiency was confirmed by immunoblotting. PRIMA-1^Met^ was purchased from Tocris Bioscience (R&D Systems, MN, USA). AZD6244 was purchased from Array BioPharma (Boulder, CO, USA).

### MTS assay

The cells were seeded (1×10^3^/well) in 96-well plates and cultured overnight. Cells were treated with different concentrations of PRIMA-1^Met^ and cell proliferation inhibition was measured by MTS assay (Promega, WI, USA) according to the manufacturer's instructions.

### Anchorage-independent cell growth assay

A total of 8,000 cells were suspended in 1 ml solidified Basal Medium Eagle containing 10% FBS and added to 0.33% top agarose with various concentrations of PRIMA-1^Met^. A base layer of 0.6% agarose was placed at the bottom of top agar. Three weeks after seeding, colonies were counted in low-power field of light microscope using the Image-Pro Plus Software (vs.4) program (Media Cybernetics).

### Kinase profiling

Kinase profile assay was performed according to Millipore's Kinase Profiler protocols. The concentration of PRIMA-1^Met^ and ATP for each kinase analysis was set at 100 uM and 10 uM, respectively.

### Molecular modeling

Briefly, the PDB files of compound PRIMA-1^Met^ was generated from the PRODRG server [37]. The X-ray crystallographic structures of MEK-1/2 (PDB code: 1S9I [38]) were downloaded from Protein Data Bank and prepared by using the Protein Preparation Wizard in Maestro v9.2. Hydrogen atoms were added to the complexes consistently with a pH of 7 and water molecules were deleted. Ligand docking the compound to MEK-1/2 monomer was accomplished using the automated docking tool AutoDock 4. Ten independent runs were carried out to yield ten AutoDock docking solutions, which were ranked by the calculated energy favorable scores. Top hits based on hydrogen-bond interactions were considered as top-ranked conformations of docked complex.

### *In vitro* kinase assay

Active MEK1 (100 ng) and its inactive substrate ERK1 (1 ug) were purchased from Millipore (Temecula, CA, USA), mixed in the presence of various doses of PRIMA-1^Met^, and incubated in 100 uM ATP and ×1 kinase buffer (10 mM MgCl_2_, 1 mM EDTA, 50 mM Tris-HCl pH 7.5, 0.01% Brij35, 1 mM DTT; Cell Signaling Technology) at 30°C for 30 min. Then the samples were resolved in ×6 SDS loading buffer. Phosphorylated ERK1 and total MEK protein level were analyzed by Western blot analysis.

### *In vitro* ATP competitive assay and pull-down binding assay

PRIMA-1^Met^-conjugated Sepharose 4B beads (GE Healthcare, Pittsburgh, PA, USA) were prepared according to the manufacturer's instruction. The ATP competition assay was conducted by incubating 300 ng active MEK1 protein with various concentrations of ATP (0, 10, 100 uM) at 4°C for 2 h, followed by incubation with 100 ul PRIMA-1^Met^-conjugated Sepharose 4B beads or Sepharose 4B beads only for another 2 h in ×1 reaction buffer (150 mM NaCl, 50 mM Tris-HCl pH 7.5, 5 mM EDTA, 0.01% NP40, 1 mM DTT, 0.02 mM phenylmethysulfonyl fluoride, 2 ug/ml bovine serum albumin and ×1 protease inhibitor mixture). For pull-down binding assay, HCT116^neg^ cell lysates (600 ug) were incubated with 100 ul PRIMA-1^Met^-conjugated Sepharose 4B beads or Sepharose 4B beads only overnight at 4°C, then the beads were washed with ×1 wash buffer (50 mM Tris-HCl pH 7.5, 150 mM NaCl, 1 mM DTT, 0.02 mM phenylmethylsulfonyl fluoride and 0.01% NP40). The proteins that bound to PRIMA-1^Met^-conjugated Sepharose 4B beads were resolved by 10% SDS-PAGE and detected by Western blot analysis.

### Measurement of ROS

HCT116^neg^ and HCT116^wt^ cells were cultured in six-well plate and grown to subconfluency. Cells were treated with PRIMA-1^Met^ in the presence or absence of NAC for indicated time. Then cells were stained with 20 uM DCFHDA for 30 min and fluorescence was analyzed using flow cytometer in the FITC channel. The fluorescence intensity of 15,000 cells was measured by WinMDI 2.8 software and normalized to vehicle group.

### Western blot analysis

Cells were lysed with RIPA buffer (150 mM NaCl, 50 mM Tris-HCl pH 8.0, 0.25% SOD, 0.1% sodium dodecyl sulfate, 1 mM EDTA, 1% NP40 and ×1 protease/phosphatase inhibitor cocktail). Lysates from mouse xenograft were prepared by homogenizing tumor mass in RIPA buffer. Proteins were separated by SDS-PAGE and transferred to PVDF membranes (Amersham Pharmacia Biotech). Membranes were blocked with 5% non-fat milk and then probed with appropriate primary antibody overnight at 4°C. Then membranes were washed in PBS three times and incubated with horseradish peroxidase-conjugated secondary antibody. Protein bands were visualized using an enhanced chemiluminescence reagent kit (Amersham). Antibodies against cleaved caspase 3, total MEK1/2, phosphorylated MEK1/2 (Ser217/221), phosphorylated ERK1/2 (Thr202/Tyr204), phosphorylated p90RSK (Thr359/Ser363) and b-actin were purchased from Cell Signaling (Beverly, MA, USA). Antibodies against total ERK1/2, total MEK1/2, TP53, p21 and Ki-67 were from Santa Cruz Biotechnology (Santa Cruz, CA, USA).

### *X*enograft mouse model

Athymic nude mice (6-9 weeks old) were provided by SLAC Laboratory Animal Co. Ltd. (Shanghai, China). Briefly, cells in logarithmic phase were harvested and suspended in cold PBS at 5×10^6^/mL. 1×10^6^ cells (in 200 μl of PBS) were injected subcutaneously into right flank of each mouse. Once the tumor volume reached around 50 mm^3^, mice were randomly divided into three groups (n=5 each): (i) vehicle group; (ii) 20 mg/kg PRIMA-1^Met^-treated group; (iii) 100 mg/kg PRIMA-1^Met^-treated group. PRIMA-1^Met^ or vehicle (PBS) was intraperitoneally injected daily for 5 days, stopped for 2 days and reinjected for additional 5 days. Tumor volume was measured twice a week based on the following formula: volume = (Length×Width^2^)/2. Mice body weight was measured and recorded once a week. Mice were euthanized on day 28 and tumor tissues were dissected for further analysis.

### Immunohistochemistry analysis

The tumor samples were fixed in 10% neutral formalin solution and embedded in paraffin. Then sections were cut into fragments of 6 um, deparaffinized and hydrated. Tissues on slides were permeabilized by 0.5% Triton X-100 and incubated overnight at 4°C with primary antibodies against Ki67 and phosphorylated ERK1/2. The slides were washed by PBS for 5 times and incubated with 1:200 dilution of secondary biotinylated antibody for 2 h at room temperature. All slides were developed using horseradish peroxidase-streptavidin (Santa Cruz Biotechnology, CA, USA). Images were taken under microscope and signal densities were analyzed using the Image-Pro Plus Software (Media Cybernetics).

### Statistical analysis

All data were presented as mean values ± SD or SE as indicated. The Student's t test (two groups only) or a one-way ANOVA (more than two groups) was used for the comparison. *p* < 0.05 was accepted as statistical significance.

## SUPPLEMENTARY FIGURES AND TABLE


