# Supplementary material for: PRIMA-1Met suppresses colorectal cancer independent of p53 by targeting MEK
Source: Oncotarget. 2016 Oct 27;7(50):83017–30. doi: 10.18632/oncotarget.12940 (PMC5347749; doi:10.18632/oncotarget.12940)
Supplement: Supplementary file 1 [file oncotarget-07-83017-s001.pdf]

PRIMA-1^{Met} suppresses colorectal cancer independent of p53 by targeting MEK

SUPPLEMENTARY FIGURES AND TABLE

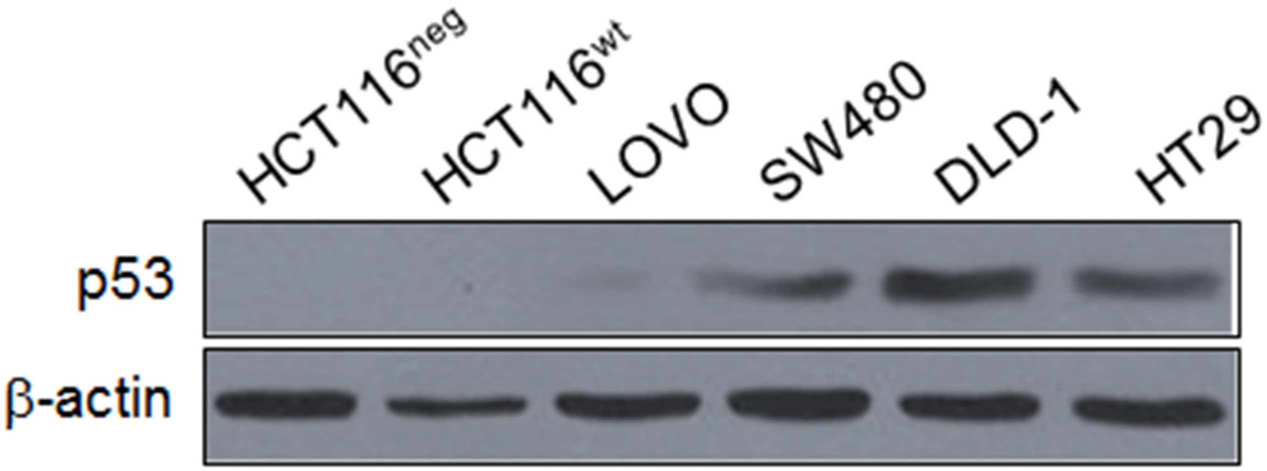

Supplementary Figure S1: p53 protein expression levels in colorectal cancer cell lines. A series of colorectal cancer cell lines representative of different p53 status, consisting of TP53^{wt} (HCT116^{wt} and LOVO), TP53^{mut} (SW480, DLD-1 and HT29) and TP53^{neg} (HCT116^{neg}), were lysated and p53 protein expression levels were determined by Western blot analysis. Actin was used as loading control.

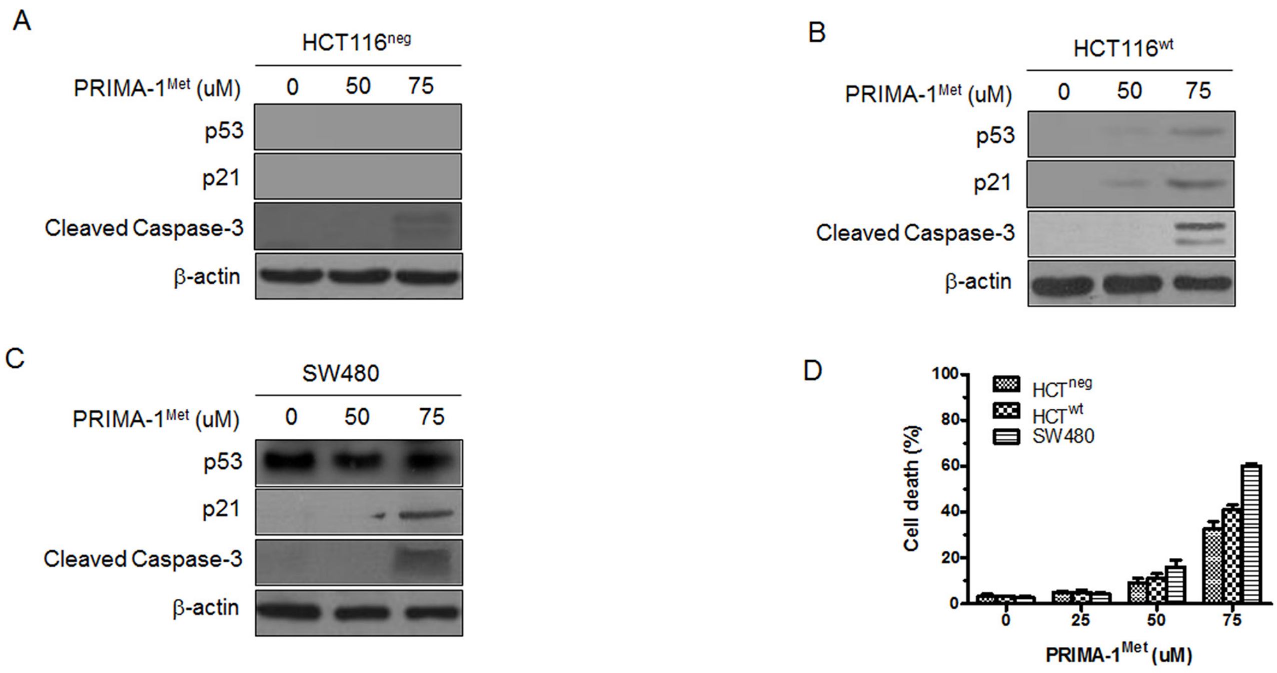

Supplementary Figure S2: PRIMA-1^{Met} induces apoptosis of colorectal cancer cells in a dose dependent manner. A-C. HCT116^{neg}, HCT116^{wt} and SW480 cells were treated with various concentrations ofPRIMA-1^{Met} (0, 50, 75 uM) for 24 h. The levels of p53, p21 and cleaved caspase-3 were detected by Western blot analysis. **D.** Colorectal cancer cells were treated with serial concentrations of PRIMA-1^{Met} (0, 25, 50, 75 uM) for 24 h. Cell death was assessed by using Apo2.7 staining kit. Data were shown as mean ± SEM (n=3).

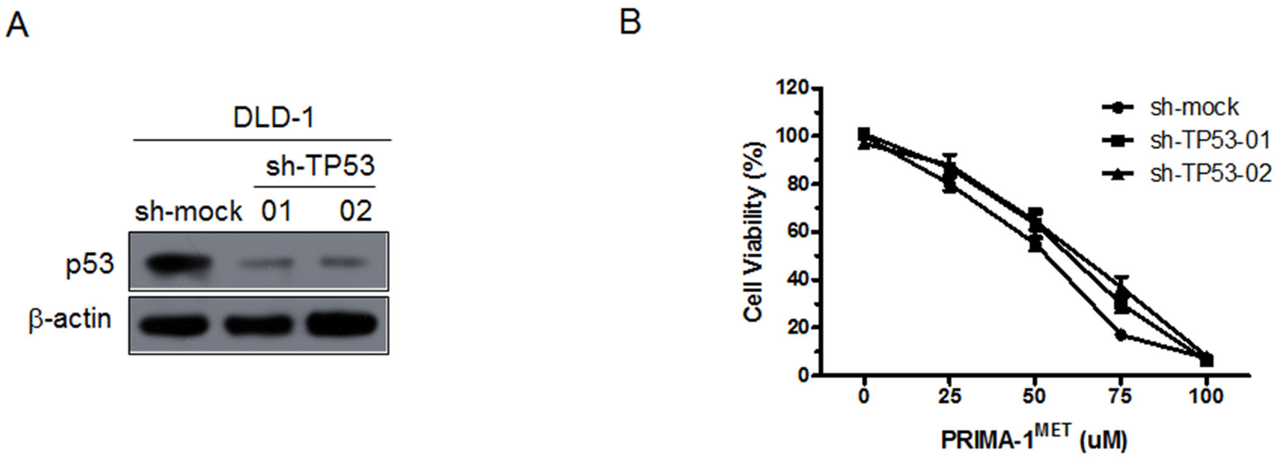

Supplementary Figure S3: PRIMA-1^{Met} inhibits the proliferation of DLD-1 colorectal cancer cells independent of mutant p53 expression level. A. DLD-1 cells were transiently transfected with sh-TP53 or sh-mock and p53 expression was analyzed by Western blot analysis. **B.** DLD-1 cells transfected with sh-mock or sh-TP53 were treated with various concentrations of PRIMA-1^{Met} for 24 h and cell viability was measured by MTS assay. Data were showed as mean ± SEM (n=3).

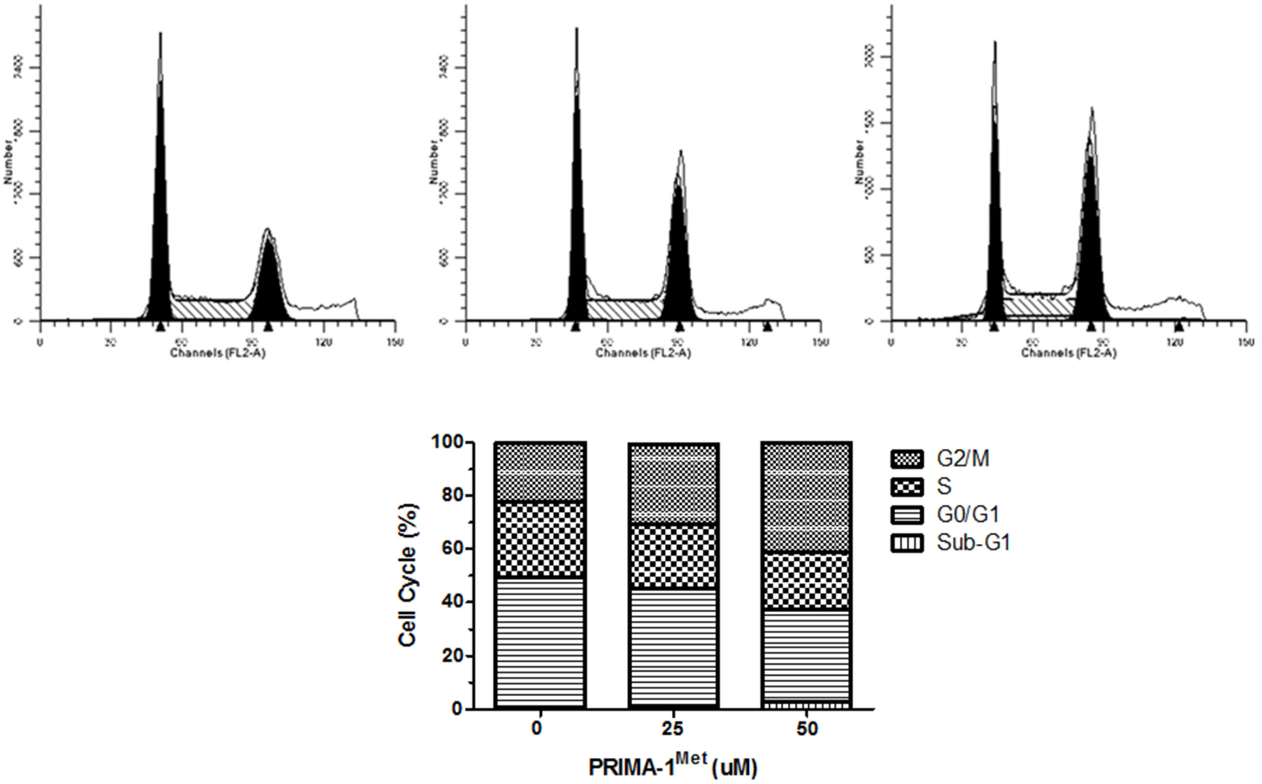

Supplementary Figure S4: PRIMA-1^{Met} induces G2/M arrest in HCT116^{neg} cells. HCT116^{neg} cells were treated with various doses of PRIMA-1^{Met} (0, 25, 50 uM) for 12 h. Cell cycle was assessed by flow cytometry. Representative images of cell cycle distribution were shown from three independent experiments.

Supplementary Table S1: The genotype of TP53 in colorectal cell lines

Cell line	Genomic profile	Mutation site	Mutation type
HCT116^{wt}	TP53 wild type	-	-
LOVO	TP53 wild type	-	-
SW480	TP53 mutated	Exon 8, codon 273 (G>A)	Arg→His
DLD-1	TP53 mutated	Exon 7, codon 241 (C>T)	Ser→Phe
HT29	TP53 mutated	Exon 8, codon 273 (G>A)	Arg→His
